# DFT Calculations on Electronic, Thermochemical and Vibrational Properties of Se_6_ Selenium Clusters as 5-Fluorouracil Drug Delivery System

**DOI:** 10.3390/biotech15020029

**Published:** 2026-03-31

**Authors:** Levi Isai Solano-González, Raúl Mendoza-Báez, Ricardo Agustín-Serrano, José Isrrael Rodríguez-Mora, Marco A. Morales

**Affiliations:** 1 Facultad de Ingeniería Química, Benemérita Universidad Autónoma de Puebla, Av. San Claudio18 Sur S/N, San Manuel, Puebla C.P. 72570, Mexico; levisai.sg@gmail.com; 2 Departamento de Química, Centro de Investigaciónde Estudios Avanzados del IPN (Cinvestav), Av. IPN 2508, Col. San Pedro Zacatenco, Ciudad de México C.P. 07360, Mexico; mendozabaezr@gmail.com; 3 Facultad de Físico Matemáticas, Benemérita Universidad Autónoma de Puebla, Av. San Claudio18 Sur S/N, San Manuel, Puebla C.P. 72570, Mexico; ricardoagustin_s@hotmail.com; 4 Laboratorio Interdisciplinario de Impresión 3D Para la Innovación Tecnológica, Benemérita Universidad Autónoma de Puebla, Av. San Claudio S/N, Cd. Universitaria, Puebla C.P. 72570, Mexico; 5 Facultad de Ingeniería, Benemérita Universidad Autónoma de Puebla, Av. San Claudio18 Sur S/N, San Manuel, Puebla C.P. 72570, Mexico; 6 Laboratorio de Investigación de AltaTecnología y Desarrollode Prototipos Patentes, Benemérita Universidad Autónoma de Puebla, Av. San Claudio S/N, Cd. Universitaria, Puebla C.P. 72570, Mexico

**Keywords:** DFT, drug delivery, 5-fluorouracil, cancer, molecular simulation

## Abstract

In this work, the electronic, thermochemical, and vibrational characterization of the drug delivery system formed by clusters of selenium (Se_6_ allotrope) and 5-fluorouracil (5-FU) are studied, based on density functional theory (DFT) calculations. Computational calculations were performed using the B3LYP functional and the 6-31G(*d*,*p*) base set, considering an aqueous medium through the CPCM solvation model. We propose evaluating two different interaction modes based on experimental observations: Se–H(N) (through the amino groups of 5-FU) and Se–O(C) (through the carbonyl oxygen of 5-FU). All complexes proved to be energetically stable, exhibiting chemisorption as their adsorption process. Analysis of adsorption energy and thermodynamic parameters indicates that both interaction pathways are equally viable, which agrees with previous experimental findings. The theoretical FT-IR spectra of these complexes also coincide with the experimental results. Furthermore, global molecular descriptors show that the stability of the selenium carrier is not affected by post-functionalization, which is desirable for more controlled drug delivery systems.

## 1. Introduction

Data from the International Agency for Research on Cancer (IARC) indicates that approximately 20 million new cases of cancer were diagnosed from 2022 to date, resulting in 9.7 million deaths from the disease. Estimates suggest that roughly one in five men or women will develop cancer during their lifetime. Demographic projections indicate that the number of new cancer cases will reach 35 million by 2050 [1]. Given this alarming reality, the development of new therapies and effective drugs for its treatment is becoming increasingly urgent. In this context, nanomaterial-based drug delivery systems have gained significant relevance. Several studies support the use of nanoparticles as delivery systems due to their structural, chemical, physical, and biological properties which make them highly functional as transport agents that can encapsulate or bind to specific drug components, thereby facilitating their more precise and efficient release [2,3,4,5,6]. However, despite their multiple benefits, these technologies still face significant challenges. Some nanoparticles, such as those of iron, gold, silver, or carbon, are not biodegradable, making them difficult to eliminate from the body once administered, and they can also present instability problems in biological environments, inhibiting their effectiveness [7,8,9,10,11]. For these reasons, current research is being directed toward finding new alternatives that offer safer drug delivery systems: biocompatible, stable, bifunctional and biodegradable. In this sense, selenium-based nanoparticles (SeNPs) have recently proven to be promising materials for these applications, being evaluated both in vitro and in vivo, and using pristine or functionalized nanoparticles [12,13,14,15,16,17,18]. In addition to their use as chemotherapy agents, SeNPs have interesting structural, electrical, magnetic, optical, antioxidant, and antibacterial properties [19,20,21,22,23,24]. Selenium is incorporated into polypeptides such as selenocysteine and seleno-methionine, two key amino acids involved in various biochemical processes, and it also stimulates the activation of immune cells [25,26,27]. Although selenium is an essential trace element that plays a key role in the regulation of redox reactions at the cellular level and is also involved in several fundamental physiological functions of the body (in form of seleno-proteins), exceeding its dosage (412 μg/day) causes harmful side effects such as selenosis [28,29,30,31]. However, the advantage of using SeNPs is that they can be as effective as other organoselenium compounds but with lower toxicity [32,33,34].

On the other hand, 5-fluorouracil (5-FU, or fluoropyrimidine) has been widely used in the treatment of skin, colon, breast, gastric, and esophageal cancer [35,36,37,38]. It is an antimetabolite, whose chemical structure resembles that of the nucleotide uracil, one of the components of RNA, and this similarity allows it to be erroneously incorporated into nucleic acids during DNA and RNA synthesis in rapidly dividing cells, inhibiting their division and growth [39,40,41]. Liu et al. [42] experimentally studied the behavior of the system composed of SeNPs and 5-FU, revealing a remarkable synergy: a significant reduction in the cytotoxicity toward human normal cells and an improvement in the cellular uptake of SeNPs in cancer cells. These complementary properties make the system a promising drug delivery strategy, thus increasing interest in its study.

In this work, the Se_6_/5-fluorouracil (Se6/5-FU) system, a carrier–drug complex, is studied based on density functional theory (DFT) calculations to evaluate the intermolecular interactions that govern this complex and its structural, electronic, thermochemical and vibrational properties. In addition, the chemical reactivity of the complexes was studied using global chemical descriptors, as well as through cohesive and solvation energies. This computational study aims to elucidate the interaction mechanisms underlying the observed synergy between SeNPs and 5-FU, thus enabling the possible development of a safer and more effective drug delivery system.

## 2. Materials and Methods

*Materials:* Two nodes of the Laboratorio Nacional de Supercómputo del Sureste de México, Puebla, México, 600 Core Hours, storage space in the/home partition up to 1 GB were used for the development of the project.

*Level of theory*: Optimization and frequency calculations were performed based on density functional theory (DFT), considering a neutral charge (Q=0) and singlet multiplicity (M=1) for all molecules and complexes studied, using the B3LYP [43,44] functional with a 6-31G(d,p) basis set, and considering aqueous medium through the CPCM (Conductor-like Polarizable Continuum Model) implicit solvation model [45,46], as implemented in the Gaussian software package in their version 09 [47]. The CPCM model has recently been used as a suitable solvation model for the study of organoselenium compounds and Se–metal coordination complexes [48,49,50]. Previous work has shown that the B3LYP/aug-cc-pVDZ, B3LYP/6-31G(d) and PBE0/6-311G(d) methods adequately describe small selenium rings (Se_n_, n = 2–8), agreeing reasonably with what is experimentally observed in the solid state [51,52,53,54,55,56]. In this work, the basis set was expanded by adding *p* functions, namely B3LYP/6-31G(d,p). However, while the B3LYP/6-31G(d,p) level of theory has some limitations compared to other functionals/basis sets, recent studies have shown that B3LYP/6-31G(d,p) is suitable for describing the electronic, chemical, and optical properties of systems containing selenium atoms [57,58,59]. We do not consider the level of theory we employ to be obsolete, although other, more rigorous functionals/basis sets can certainly be evaluated. So, this is currently beyond the scope of our work and is a topic for future research.

**Figure 1:**
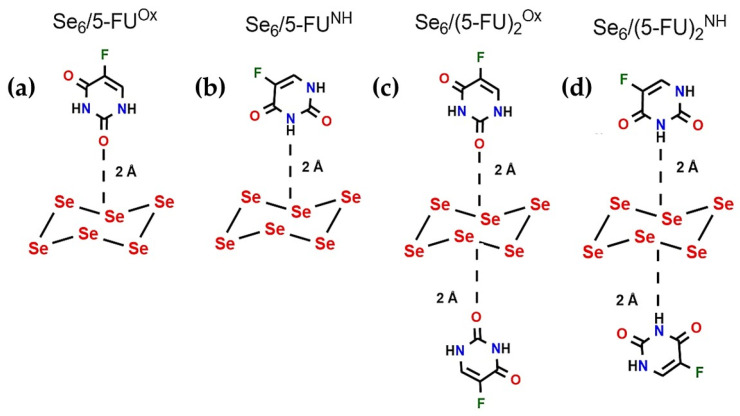
Interaction models used for the Se_6_/5-FU systems in 1:1 (**a**,**b**) and 1:2 (**c**,**d**) ratios. The initial distance was 2 Å, bringing the Se–O(C) atoms (**a**,**c**) and Se–H(N) atoms (**b**,**d**) closer together as the main interaction sites.

**Figure 2:**
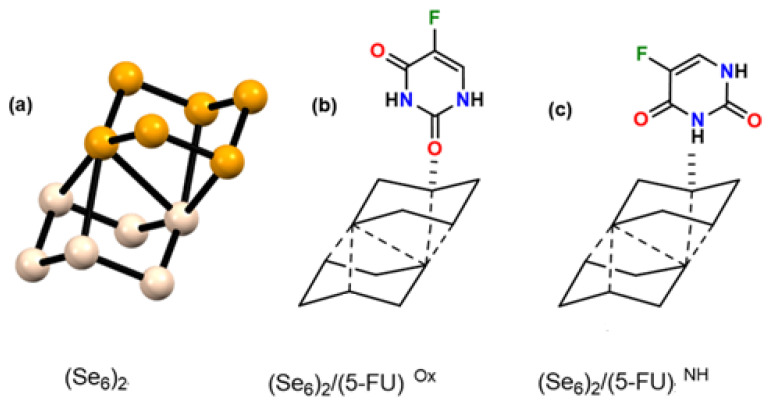
(**a**) Ball and stick representation of the optimized dimer (Se_6_)_2_. Interaction models used for the (Se_6_)_2_/(5-FU) systems where the initial distance was 2 Å, bringing the (**b**) Se–O(C) atoms and (**c**) Se–H(N) atoms closer together as the main interaction sites.

*Computational molecular models*: The Se_6_ ring-like cluster with D_3d_ symmetry was used as the drug–carrier complex, as this conformer has lower energy compared to Se_6_(D_2_) and Se_6_(C_2v_) [52,60]. Furthermore, the Se_6_(D_3d_) cluster is the one that has been experimentally observed. We considered two different interaction models between Se_6_ and the 5-FU molecule: (a) interaction between Se atoms and the carbonyl oxygen (Se_6_/5-FU^Ox^), and (b) interaction between Se atoms and the amino group -NH belonging to the pyrimidine ring (Se_6_/5-FU^NH^) (see Figure 1). This was based on the experimental findings of Liu et al. [42], who suggested Se–O and Se–N bonds. We only considered the di-keto tautomer of 5-FU, since it is its most stable structure [61] and, in addition, the experiments of Liu et al. [42] were carried out at pH = 5.4–7.4, which would not favor the keto-enol tautomer. It was also evaluated whether a single Se_6_ cluster allows the carrying of two 5-FU molecules (see Figure 1c,d). Furthermore, we analyzed the effect of a larger selenium cluster on 5-FU adsorption. To do this, we designed a Se_6_ dimer, denoted as (Se_6_)_2_, which interacted again with one 5-FU molecule through Se–O(C) and Se–N(H) approaches (see Figure 2). It is worth mentioning that all optimizations were performed in an aqueous medium (CPCM, water). The (Se_6_)_2_ dimer shown in Figure 2a was the lowest energy conformer found, with an offset, stacked ring structure.

The reactivity of these systems was studied through global molecular descriptors (chemical potential μ, global hardness η, electrophilicity index ω, Equations (1)–(3)) derived from Koopmans’ theorem, ref. [62] which states that

(1) $$\mu ={\left(\frac{\partial E}{\partial N}\right)}_{\nu \left(r\right)}=-\left(\frac{I+A}{2}\right)$$

(2) $$\eta ={\left(\frac{\partial^{2}E}{\partial N^{2}}\right)}_{\nu \left(r\right)}=\frac{I-A}{2}$$

(3) $$\omega =\frac{\mu^{2}}{2\eta }$$

where E is the total energy, N is the number of electrons, ν(r) is the external potential of the system, I is the ionization potential and A is the electron affinity [63,64,65]. All the aforementioned descriptors are calculated from the energies of frontier molecular orbitals (HOMO and LUMO), since $I=-E_{HOMO}$ and $A=-E_{LUMO}$. The energy difference between the frontier molecular orbitals, $E_{gap}$ (Equation (4)), was also calculated. As the stability of the structures decreases and chemical reactivity increases, the $E_{gap}$ value decreases.

(4) $$E_{gap}=\left|E_{HOMO}-E_{LUMO}\right|$$

On the other hand, the stability of the systems was evaluated by calculating the cohesive energy ($E_{coh}$, Equation (5)), which is defined as the energy required to break all the bonds of a molecule down to its individual atoms (eV/atom units). $E_{complex}$ is the total energy of the complex formed (e.g., Se_6_/5-FU), the coefficient $n_{i}$ corresponds to the number of i-atoms (Se, C, H, N, O, F), and $E_{i}$ is the total energy of each i-atom. A more stable system is characterized by lower $E_{coh}$ values [66,67,68].

(5) $$E_{coh}=\frac{E_{complex}-\left(n_{i}E_{i}\right)}{\sum n_{i}}$$

To quantify the interaction between Se_6_ and 5-FU, the adsorption energy (Equation (6)) was calculated using

(6) $$E_{ads}=E_{complex}-\left({nE}_{{\left({Se}_{6}\right)}_{n}}+mE_{{\left(5FU\right)}_{m}}\right)$$

The term $E_{{\left({Se}_{6}\right)}_{n}}$ is the total energy of Se_6_ (if n=1) or (Se_6_)_2_ (if n=2), while $E_{{\left(5FU\right)}_{m}}$ is the total energy of the number of drug molecules being adsorbed (m=1,2). There is a classification based on $E_{ads}$ that defines physisorption when $E_{ads}>-0.5$ eV or chemisorption when $E_{ads}<-0.5$ eV [69,70]. Therefore, a more negative $E_{ads}$ value indicates a stronger interaction between Se_6_ and 5-FU.

Vibrational frequency calculations were performed (at 298.15 K) to find that the molecules relax with local minima, demonstrating that all systems exhibit non-imaginary frequencies. The normalized transmittance (T) of the IR spectra was obtained from the molar absorptivity (Equation (7)).

(7) $$T(\%)=100\cdot \left(1-\frac{\epsilon^{i}}{\epsilon^{max}}\right)$$

where $\epsilon^{i}$ is each molar absorptivity (M^−1^cm^−1^) value obtained from the frequency calculations and $\epsilon^{max}$ is the maximum molar absorptivity found. The frequency calculations were also used to calculate thermodynamic parameters such as enthalpy (∆H), Gibbs free energy (∆G) and entropy (∆S). By utilizing the internal electronic energy Ɛ_0_ combined with the thermal corrections for enthalpy H_corr_ and Gibbs free energy G_corr_, the reaction values can be determined. Since the computational output provides the sum of electronic and thermal enthalpies, the total enthalpy of reaction is obtained by calculating the difference between the sums of these values for products and reactants [71].

(8) $${∆}_{r}H^{{}^{\circ}}\left(298.15\;K\right)=\sum_{products}(\varepsilon_{0}+H_{corr})-\sum_{reactants}(\varepsilon_{0}+H_{corr})$$

(9) $${∆}_{r}G^{{}^{\circ}}\left(298.15\;K\right)=\sum_{products}(\varepsilon_{0}+G_{corr})-\sum_{reactants}(\varepsilon_{0}+G_{corr})$$

(10) T∆S=∆H−∆G

## 3. Results

### 3.1. Structural Properties

The cyclic Se_6_ molecule showed a chair-type conformation, D_3d_ symmetry, with an average Se–Se bond length of 2.355 Å, in accordance with what has been reported experimentally and theoretically [51,52,53,54,55,56]. The average bond angle is 100.25° and the dihedral angles are 77.5°. The dimeric cluster (Se_6_)_2_ exhibits slightly longer Se–Se bond lengths compared to Se_6_, of 2.363 Å. However, its bond angles show values in a range of 99.61–103.00°, indicating that the Se_6_ rings deform upon dimerization. (Se_6_)_2_ is arranged in an off-center stacked structure, with a distance of 3.970 Å between the two Se_6_ rings. Although (Se_6_)_2_ is a cluster-type structure, the intermolecular Se–Se bond lengths are longer (3.165 Å) than the intramolecular ones (2.363 Å). On the other hand, the optimized 5-FU molecule showed bond lengths consistent with those reported experimentally in the solid state, with distances C=O 1.223 Å, C–F 1.344 Å and C=C 1.348 Å for the calculated structure, and C=O 1.222 Å, C–F 1.343 Å and C=C 1.378 Å for those reported experimentally [72].

### 3.2. Electronic Properties

#### 3.2.1. Frontier Molecular Orbitals and Global Molecular Descriptors

**Figure 3:**
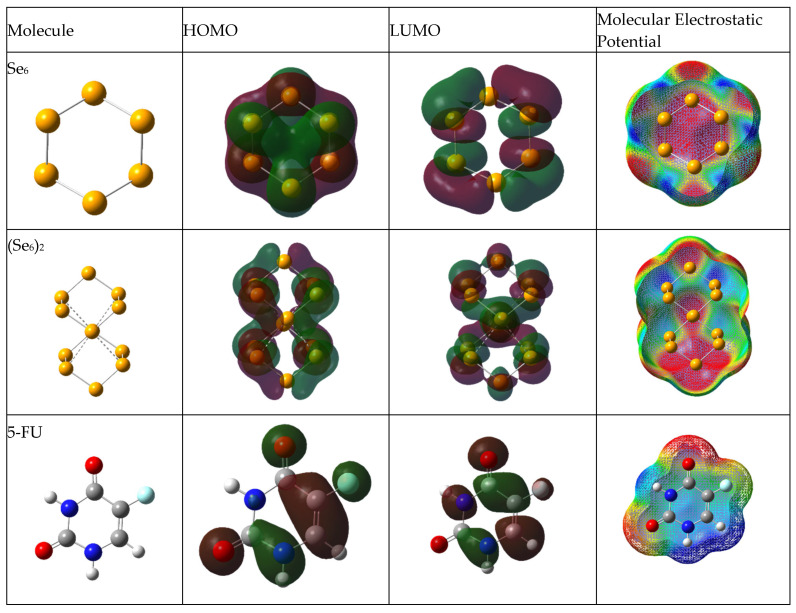
Optimized geometries of Se_6_ (Se_6_)_2_ and 5-FU molecules, HOMO and LUMO orbitals, and molecular electrostatic potential, obtained with B3LYP/6-31G(d,p) using CPCM solvation model (water).

**Figure 4:**
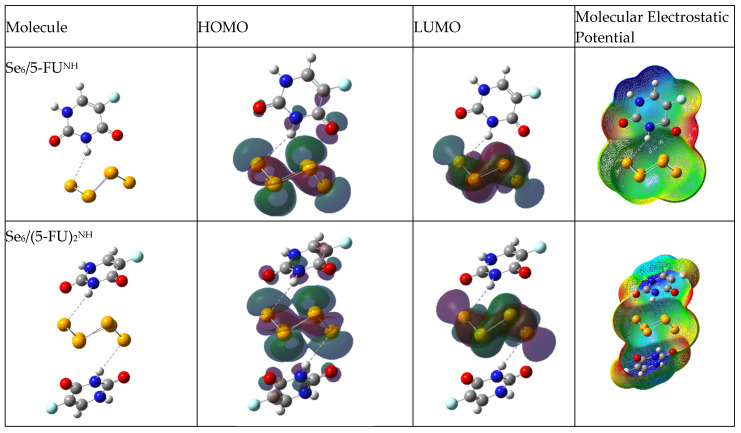

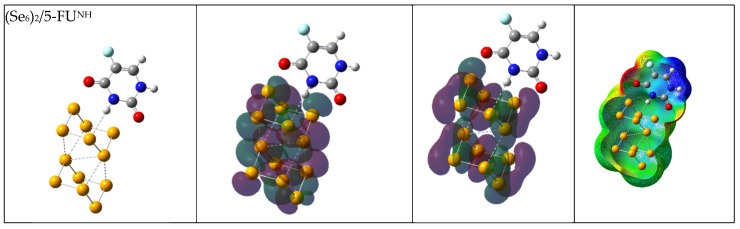
Optimized geometries of Se_6_/5-FU^NH^, Se_6_/(5-FU)_2_^NH^ and (Se_6_)_2_/5-FU^NH^ complexes, HOMO and LUMO orbitals, and molecular electrostatic potential, obtained with B3LYP/6-31G(d,p) using CPCM solvation model (water).

**Figure 5:**
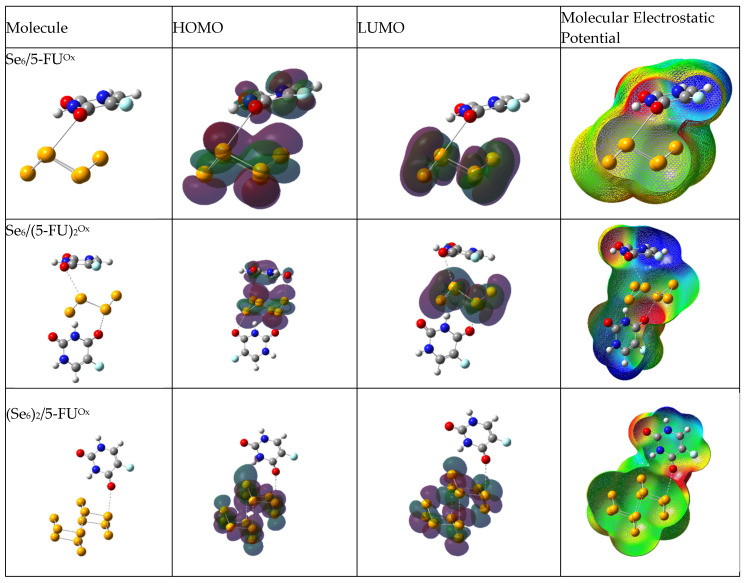
Optimized geometries of Se_6_/5-FU^Ox^, Se_6_/(5-FU)_2_^Ox^ and (Se_6_)_2_/5-FU^Ox^ complexes, HOMO and LUMO orbitals, and molecular electrostatic potential, obtained with B3LYP/6-31G(d,p) using CPCM solvation model (water).

The density distributions of the HOMO and LUMO molecular orbitals, and the mapping of the molecular electrostatic potential (MEP), are shown in Figure 3, Figure 4 and Figure 5 for aqueous medium. Supplementary Materials Figures S1–S5 present the density distributions of the HOMO and LUMO molecular orbitals and the mapping of the molecular electrostatic potential (MEP) for vacuum medium. For electron density distributions in aqueous medium, the HOMO and LUMO of (Se_6_)_2_ are distributed throughout the cluster, showing the interaction between both rings. Figure 4 shows the frontier molecular orbitals (FMOs) of the Se/5-FU complexes connected by Se–H(N), namely Se_6_/5-FU^NH^, Se_6_/(5-FU)_2_^NH^ and (Se_6_)_2_/5-FU^NH^. For those complexes with Se_6_, the HOMO is distributed on both Se_6_ and the 5-FU molecule. However, when the adsorption is carried out by the (Se_6_)_2_ dimer, the HOMO is located only on (Se_6_)_2_. In the case of LUMO, it is located at (Se_6_)_n_ in all cases. This indicates that the selenium cluster is the most reactive site of the drug–carrier complex, thus preventing possible reactions on 5-FU. Figure 5 shows the frontier molecular orbitals (FMOs) of the Se/5-FU complexes connected by Se–O(C), namely Se_6_/5-FU^Ox^ and Se_6_/(5-FU)_2_^Ox^. Analogously to what was described above, HOMO and LUMO again fall on the (Se_6_)_n_-moiety. Regarding the MEPs, it is observed that in all complexes, the neutral potential region (green) lies on the selenium units, while the negative potential region (red) is located in the vicinity of the carbonyl oxygens of the 5-FU molecule. Furthermore, the region of lowest electron density (blue) is located on the N–H and C–H hydrogens adjacent to the fluorine atom. It is worth noting that the 5-FU MEP is not apparently disturbed after its adsorption on Se_6_, suggesting a retention of its electronic distribution even after its functionalization.

The stability and reactivity of the complexes are determined using global molecular descriptors, such as chemical potential (μ), global hardness (η), and electrophilicity index (ω), as well as the HOMO−LUMO energy gap ($E_{gap}$), cohesive energy ($E_{coh}$), and adsorption energy ($E_{ads}$). Table 1 and Table 2 summarize these quantities. Global hardness (η) and chemical potential (μ) are involved within the ω descriptor, the electrophilicity index, which measures a molecule’s ability to accept electrons in an electron-rich environment [64,65]. Therefore, a good electrophile (high ω value) will be characterized by high μ values and low η values. From Table 1, we observe that the $E_{gap}$ value decreases from 4.112 to 2.995 eV as the Se–unit grows from Se_6_ to (Se_6_)_2_. This is mainly due to a destabilization of the HOMO, increasing its energy, while the LUMO remains almost invariant after cluster growth.

This also impacts the slight increase in electrophilicity of (Se_6_)_2_ (ω = 5.476 eV) compared to Se_6_ (ω = 4.872 eV). This suggests that, if the trend continues, larger SeNPs would be disadvantaged due to their greater chemical reactivity and instability. Regarding the formation of complexes with Se_6_, it is observed that the adsorption of 5-FU does not alter the reactivity of the system, since the differences in the values of the molecular descriptors are negligible between pristine Se_6_ and Se_6_/5-FU complexes (ω = 4.7–4.9 eV). Furthermore, there are no differences due to the way in which Se_6_ and 5-FU interact, i.e., whether it is through Se–H(N) or Se–O(C). The complexes formed based on (Se_6_)_2_ exhibit higher electrophilicity index values, thought this is not due to the adsorption of 5-FU but to the electrophilic nature of the dimer, as discussed above.

#### 3.2.2. Adsorption Energy and Cohesive Energy of Complexes

It is interesting to note that, in addition to the fact that the adsorption of 5-FU does not destabilize the Se cluster, there is a strong interaction between Se_6_ or (Se_6_)_2_ and 5-FU, as shown by the $E_{ads}$ values summarized in Table 2. All values correspond to chemisorption, except for the Se_6_/5-FU^NH^ complex (−0.46 eV), with the Se_6_/(5-FU)_2_^NH^ complex having the highest adsorption energy (−1.04 eV). If we compare the complexes that adsorb a single 5-FU molecule (Se_6_/5-FU^Ox^, Se_6_/5-FU^NH^, (Se_6_)_2_/5-FU^Ox^ and (Se_6_)_2_/5-FU^NH^), we will observe very similar $E_{ads}$ values, suggesting that: (1) it would be indifferent to use Se_6_ or (Se_6_)_2_ as a 5-FU carrier in 1:1 ratio, and (2) both proposed interaction pathways are equally energetically favorable. Interestingly, complexes with a 1:2 ratio (Se_6_/(5-FU)_2_^Ox^, Se_6_/(5-FU)_2_^NH^) exhibit the strongest interaction, showing that the adsorption of a second 5-FU molecule favors not only the adsorption energy but also the stability of the complex, since these 1:2 complexes also exhibit the lowest cohesive energy. We also studied the (Se_6_)_2_/(5-FU)_2_^Ox^ and (Se_6_)_2_/(5-FU)_2_^NH^ systems but unfortunately the optimizations did not converge in both cases. Further computational calculations of systems with a larger number of 5-FU molecules are needed to determine the maximum number of 5-FU molecules that a Se_6_ cluster can carry. Furthermore, basis set superposition error (BSSE) methods were applied for the half-vacuum case, and the Boys–Bernardi correction [73] was performed to address energy errors that can occur in a system based on non-covalent interactions, such as the Se6/5-FU system. This correction involves reverting the substrate atoms to ghost atoms and the absorbate to the ground and vice versa states, as shown in Figure S5. The results of the calculations can also be seen in Tables S2 and S3 of the Supplementary Materials. Single-point calculations applying the Boys–Bernardi correction in the presence of an implicit solvent were performed using explicit fragment calculations with ghost atoms [73]. The corrected Boys–Bernardi energy value (BSSE) is −0.4709 eV, while the uncorrected value is −0.4643 eV. This corresponds to a difference of only 0.0066 eV (~0.15 kcal/mol).

#### 3.2.3. Vibrational Analysis

**Figure 6:**
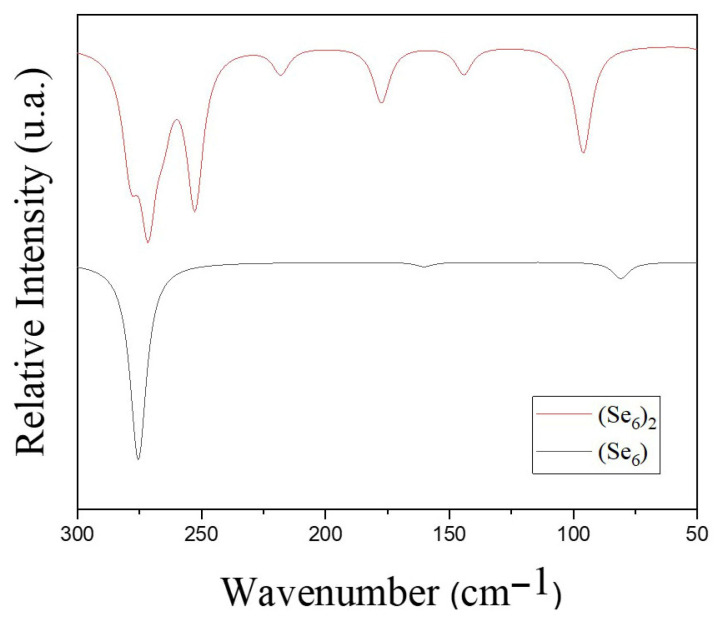
IR spectra of (Se_6_)_2_ (red spectrum) and Se_6_ (black spectrum) obtained with B3LYP/6-31G(d,p) using CPCM solvation model (water).

**Figure 7:**
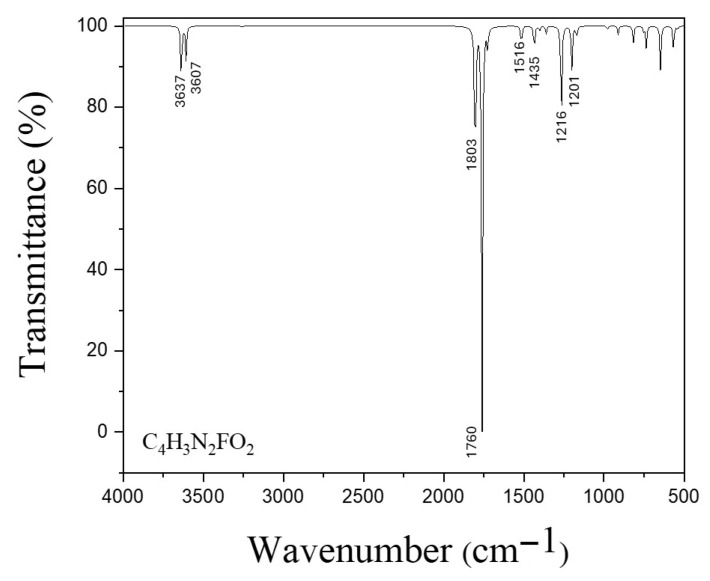
IR spectra of 5-FU obtained with B3LYP/6-31G(d,p) using CPCM solvation model (water).

**Figure 8:**
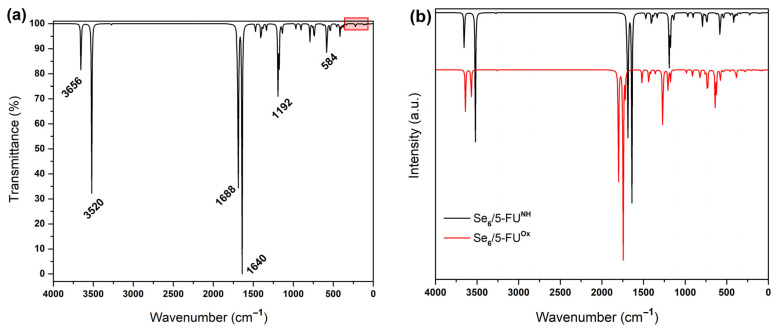
IR spectra of (**a**) Se_6_/5-FU^NH^ with the 100 cm^−1^ to 500 cm^−1^ region shaded in red corresponding to Se_6_ and magnified in Figure 7; (**b**) Se_6_/5-FU^NH^ (black line) and Se_6_/5-FU^Ox^ (red line) obtained with B3LYP/6-31G(d,p) using CPCM solvation model (water).

**Figure 9:**
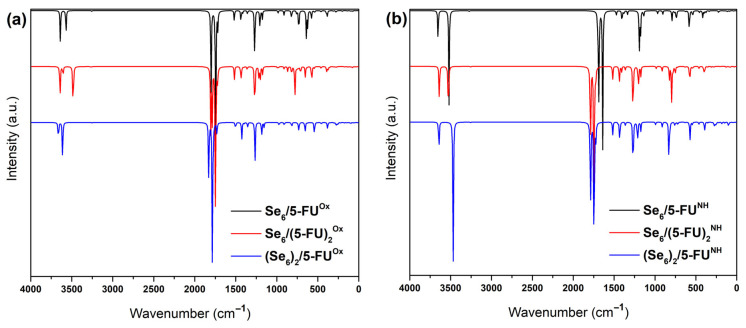
(**a**) IR spectra of (Se_6_)_2_/5-FU)^Ox^ (blue), Se_6_/(5-FU_2_)_2_^Ox^ (red), Se_6_/5-FU^Ox^ (black), and (**b**) (Se_6_)_2_/5-FU) ^NH^ (blue), Se_6_/(5-FU_2_)_2_^NH^ (red), Se_6_/5-FU^NH^ (black spectra); all models obtained with B3LYP/6-31G(d,p) using CPCM solvation model (water).

Figure 6, Figure 7, Figure 8 and Figure 9 show the calculated IR spectra for the molecules and complexes studied in this work. The frequency calculations have shown that all the systems studied exhibit non-imaginary frequency values, indicating that the structures reached global minima. First, it is observed that the IR spectrum of Se_6_ and (Se_6_)_2_ (see Figure 6) shows low wavenumber values (<300 cm^−1^), which is expected due to the large mass of the Se–atoms. (Se_6_)_2_ shows a greater number of peaks due to intermolecular Se–Se interactions. The calculated IR spectrum for 5-FU is shown in Figure 7, where peaks are observed at 1803 and 1760 cm^−1^ corresponding to C=O stretching, while experimentally they are reported at 1723 and 1672 cm^−1^, as well as a peak at 1266 cm^−1^ corresponding to C–N stretching [42]. The N–H stretching is at peak at 3637 cm^−1^. From the IR spectra of the Se_6_/5-FU^Ox^ and Se_6_/5-FU^NH^ complexes (see Figure 8), we can observe that there is a *redshift* of the peaks corresponding to the C=O and C–N vibrations, the same finding reported experimentally by Liu et al. [42], where they suggest that this *redshift* is due to the possible formation of Se–N and Se–O bonds, with which our models would agree very well. Additionally, we also observed the redshift of the peak corresponding to the N–H stretching, indicating the interaction of Se–H(N) hydrogen bonds and not just Se–N bonds.

Figure 9 illustrates: (a) models showing interactions through Se–(H)N connections, and (b) models involving Se–O(C) interactions. The vibrational frequency analysis reveals a systematic shift in the signals toward lower wavenumbers, along with an increase in the intensity of characteristic C=O and N–H vibrational peaks as the number of 5-FU molecules in the system increases.

## 4. Thermochemistry

Table 3 shows the changes in the thermodynamic parameters of the reactions that give rise to the drug–carrier complexes; that is, the reaction enthalpy (∆H), Gibbs free energy (∆G) and entropy (∆S), considering conditions at 1 atm and 298 K, shown in Table S4 of the Supplementary Materials, also presents the values of the thermochemical parameters for a medium vacuum. An important aspect to consider in IR calculations, and due to the nature of Se_6_ and (Se_6_)_2_, is that they exhibit vibration modes below −100 cm^−1^. This can cause errors when estimating Gibbs free energy values calculated within the standard rigid-rotor harmonic oscillator (RRHO) approximation. To evaluate this effect, we performed a quasi-harmonic correction for a representative system, Se_6_/5-FUNH, using a frequency cutoff of 100 cm^−1^. This approach replaces low-frequency modes with a minimum threshold, thereby reducing the artificial entropic contribution associated with very soft vibrations. The correction was carried out using the GoodVibes program [74], which implements the quasi-harmonic approximation following the Grimme entropy treatment and Head-Gordon enthalpy correction [75]. The uncorrected Gibbs free energy change (ΔG) was found to be 15.9657 kcal/mol (as shown in the Supplementary Materials, Table S5, entry 4), while the quasi-harmonic corrected value is 15.8653 kcal/mol, corresponding to a difference of only 0.10 kcal/mol. This very small deviation indicates that low-frequency vibrational modes have a negligible impact on the computed thermodynamic quantities in this system. The ∆H values are negative for all reactions in Table 3, indicating that the formation of the molecular complexes is enthalpically favorable and therefore an exothermic process, except for the Se_6_/5-FU^NH^ complex (∆H = 3.52 kcal/mol) which also exhibited the lowest adsorption energy (see above, Table 2). Only the (Se_6_)_2_/5-FU^NH^ complex would exhibit a spontaneous chemisorption process due to its negative ∆G value.

## 5. Discussion

In this study, our results of the molecular structure for the Se_6_/5-FU^Ox^ are consistent with previously reported theoretical and experimental results [51,52,53,54,55,56,68]. Furthermore, Se_6_ and its dimer (Se_6_)_2_ are electrophilic, a result of the molecular descriptors and a property of a stable molecular transport system that releases the drug when needed without altering the chemical properties of the 5-FU^Ox^ drug. In addition, we have discovered a new molecular structure for the Se_6_/5-FU^NH^ system, which doubles the possibility of experimentally obtaining a new anticancer drug delivery system. FTIR spectra of all involved molecules were also calculated, with emphasis on the characteristic peaks of the drug-releasing system for its identification and experimental verification. Finally, our results on the thermochemistry of the 5-FU^Ox^ (or 5-FU^NH^) drug-releasing molecular system show that minimal energy at surround temperature is required to their obtention. This implies the opening of an approach new to the molecular level in aqueous phase where the anticancer drug (5-FU^Ox^ or 5-FU^NH^) is released into the cancer cells, while the molecular transport medium (Se_6_ or (Se_6_)_2_) is absorbed by the host in the appropriate doses [2,20,42]. Furthermore, in the calculations shown with other functional groups and bases with dispersion correction (see Appendix A and Table S1 of the Supplementary Materials), the surprising physisorption result of our drug delivery system should have a higher release efficiency of 5-FU^Ox^ or 5-FU^NH^ from the transport medium (Se_6_ or (Se_6_)_2_) through water hydrolysis with interactions of the H and O bonds of the Se_6_/5-FU^NH^ or 5-FU^Ox^ molecules. This point warrants further study. Moreover, our theoretical results show that it is feasible to generate the system in the laboratory by mixing the reactants 5-FU and Se6 at room temperature in a 50% 5-FU–50% Se_6_ concentration. Given the simplicity of obtaining the new drug delivery system, the next step is to determine the minimum and maximum doses in in vitro experiments with cancer cells [42]. Once the ideal dose is calculated, the new 5-FU drug delivery system in fluid phase should be tested in rat biomodels with some type of cancer [42]. Based on the results obtained, this inspires further studies on non-covalent interactions to determine precisely which types of interaction forces predominate in the system. With this precise information, a novel and potentially significant molecular drug delivery system can be scaled up, ready for in vitro and in vivo experimental and clinical trials.

## 6. Conclusions

In this work, we studied the electronic, thermochemical, and vibrational properties of a drug delivery system composed of selenium clusters and 5-fluorouracil (5-FU) molecules using quantum chemistry calculations via the DFT/B3LYP/6-31G(d,p) method in aqueous media. The results show that the stability of the Se–carrier unit is not affected post-functionalization, which is very important for its use in biological systems. The adsorption energy values show that chemisorption between 5-FU and Se_6_ occurs through both Se–H(N) and Se–O(C) interactions, consistent with experimental observations [42,51,52,53,54,55,56,68]. Similarly, calculated IR spectroscopy studies show findings analogous to those reported experimentally. This shows that we can study large and complex drug delivery systems at the molecular level (and using quantum chemistry methods) even considering only small clusters and molecules instead of nanoparticles of even hundreds of nanometers, which is computationally very exhaustive. This work motivates further study of other experimentally evaluated drug transport systems to understand the molecular mechanisms behind these drug–carrier interactions.

## Figures and Tables

**Table 1:** Optimized Total Energy ($E_{T}$), Energy of the Frontier Molecular Orbitals ($E_{HOMO}$ and $E_{LUMO}$), Molecular Gap Energy (Eg), and global molecular descriptors (η, μ, ω) of all molecules and complexes (in aqueous media). All values in eV.

Molecule	$E_{T}$	$E_{HOMO}$	$E_{LUMO}$	$E_{gap}$	I	A	μ	η	ω
Se_6_	−391,746.99	−6.5318	−2.4199	4.1119	6.5318	2.4199	−4.4759	2.0560	4.8720
(Se_6_)_2_	−783,495.39	−5.5476	−2.5524	2.9952	5.5476	2.5524	−4.0500	1.4976	5.4764
5-FU	−13,988.28	−6.6137	−1.2327	5.3811	6.6137	1.2327	−3.9232	2.6905	2.8603
Se_6_/5-FU^Ox^	−405,735.79	−6.3865	−2.4027	3.9837	6.3865	2.4027	−4.3946	1.9919	4.8479
Se_6_/(5-FU)_2_^Ox^	−419,724.45	−6.3811	−2.3704	4.0107	6.3811	2.3704	−4.3757	2.0053	4.7740
(Se_6_)_2_/5-FU^Ox^	−797,484.23	−5.5326	−2.5375	2.9952	5.5326	2.5375	−4.0350	1.4976	5.4360
Se_6_/5-FU^NH^	−405,735.74	−6.4452	−2.3595	4.0858	6.4452	2.3595	−4.4024	2.0429	4.7435
Se_6_/(5-FU)_2_^NH^	−419,724.60	−6.2937	−2.3326	3.9612	6.2937	2.3326	−4.3131	1.9806	4.6964
(Se_6_)_2_/5-FU^NH^	−797,484.23	−5.5326	−2.5375	2.9952	5.5326	2.5375	−4.0350	1.4976	5.4360

**Table 2:** Adsorption energy ($E_{ads}$) and cohesion energy ($E_{coh}$) of all complexes. All values in eV.

Complex	$E_{ads}$	$E_{coh}$
Se_6_/5-FU^Ox^	−0.5207	−5.7796
Se_6_/(5-FU)_2_^Ox^	−0.8971	−6.0800
(Se_6_)_2_/5-FU^Ox^	−0.5554	−5.4579
Se_6_/5-FU^NH^	−0.4643	−5.7764
Se_6_/(5-FU)_2_^NH^	−1.0403	−6.0848
(Se_6_)_2_/5-FU^NH^	−0.5554	−5.4579

**Table 3:** Enthalpy, Gibbs free energy and entropy (kcal/mol), and dipole moment (Debyes) of the molecular complexes.

Reaction	ΔH	ΔG	TΔS	$\vec{µ}(D)$
(Se_6_ + 5-FU→Se_6_/5-FU) ^Ox^	−10.7956	0.73920	−11.5348	5.5515
(Se_6_ + 2-5-FU→Se_6_/(5-FU)_2_) ^Ox^	−18.2693	3.1318	−21.4012	3.3880
(2Se_6_ + 5-FU→(Se_6_)_2_/5-FU) ^Ox^	−3.1839	8.5535	−11.7375	4.2108
(Se_6_ + 5-FU→Se_6_/5-FU) ^NH^	3.5215	15.9657	−12.4441	6.0192
(Se_6_ + 2-5-FU→Se_6_/(5-FU)_2_) ^NH^	−21.3522	0.3093	−21.6616	4.2590
(2Se_6_ + 5-FU→(Se_6_)_2_/5-FU) ^NH^	−10.8665	−2.1222	−8.7443	5.5568

## Data Availability

The original contributions presented in this study are included in the article/Supplementary Material. Further inquiries can be directed to the corresponding authors.

## Supplementary Materials

The following supporting information can be downloaded at https://www.mdpi.com/article/10.3390/biotech15020029/s1. This file contains all the information regarding calculations in gaseous media and BSSE correction.

## Abbreviations

Se	Selenium
Se_6_	Selenium-6
CSe_6_	Cluster Selenium-6
5-FU	5-fluorouracil
**Se_6_/5-FU^Ox^**	Selenium-Oxygen
**Se_6_/5-FU ^NH^**	Selenium-Hydrogen
MAM	Marco A. Morales
RMB	Raúl Mendoza-Báez
LISG	Levi Isai Solano-González
RAS	Ricardo Agustín-Serrano
JIRM	José Isrrael Rodríguez-Mora

## Appendix A

We have recalculated the Se_6_/5-FU^NH^ and Se_6_/5-FU^Ox^ systems with different functionals (with dispersion corrections: B3LYP-D3 and wB97XD) and more robust basis sets (def2SVP and 6-311G(d,p)).

B3LYP-D3/def2SVP				wB97X-D/def2SVP			
	Energy				Energy		
Molecule	hartree	eV	E_ads (eV)	Molecule	hartree	eV	E_ads (eV)
5-FU	−513.702911	−13,978.5749		5-FU	−513.522936	−13,973.6775	
Se6	−14,407.9045	−392,059.239		Se6	−14,408.0114	−392,062.147	
Se_6_/5FU^NH^	−14,921.6187	−406,038.119	−0.30523024	Se_6_/5FU^NH^	−14,921.5409	−406,036.002	−0.1777994
Se_6_/5FU^Ox^	−14,921.6205	−406,038.17	−0.35568018	Se_6_/5FU^Ox^	−14,921.5414	−406,036.017	−0.1924931
B3LYP-D3/6-311G(d,p)				wB97X-D/6-311G(d,p)			
	Energy				Energy		
Molecule	hartree	eV	E_ads (eV)	Molecule	hartree	eV	E_ads (eV)
5-FU	−514.221016	−13,992.6732		5-FU	−514.034428	−13,987.5959	
Se6	−14,409.4567	−392,101.477		Se6	−14,409.5574	−392,104.215	
Se_6_/5FU^NH^	−14,923.6875	−406,094.416	−0.2662363	Se_6_/5FU^NH^	−14,923.5972	−406,091.957	−0.146724
Se_6_/5FU^Ox^	−14,923.6889	−406,094.454	−0.303788	Se_6_/5FU^Ox^	−14,923.597	−406,091.953	−0.14283272

The results of the adsorption energy refinement show that in all cases, the adsorption energy values decrease compared to those obtained with B3LYP/6-31G(d,p). However, they remain negative in all cases, indicating a favorable adsorption process. Furthermore, in most cases (except for wB97XD/6-311G(d,p)), the Se_6_/5-FU^Ox^ system continues to exhibit the highest (most negative) adsorption value, as observed with B3LYP/6-31G(d,p). Finally, we observed that the wB97XD functional yields considerably lower adsorption energy values compared to B3LYP/6-31G(d,p). So, as mentioned previously, a more detailed evaluation of the influence of the level of theory employed can be addressed in future work.
