# Supplementary material for: DFT Calculations on Electronic, Thermochemical and Vibrational Properties of Se6 Selenium Clusters as 5-Fluorouracil Drug Delivery System
Source: BioTech (Basel). 2026 Mar 31;15(2):29. doi: 10.3390/biotech15020029 (PMC13108189; doi:10.3390/biotech15020029)
Supplement: Supplementary file 1 [file biotech-15-00029-s001.zip › biotech-4176015-supplementary.pdf]

Figure S1. Optimized geometries of Se_6, $(Se_6)_2$ and 5-FU molecules, HOMO and LUMO orbitals, and molecular electrostatic potential, obtained with B3LYP/6-31G(d,p) in a vacuum or in a gaseous medium.

Molecule	HOMO	LUMO	MEP
5-FU 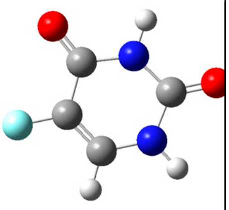	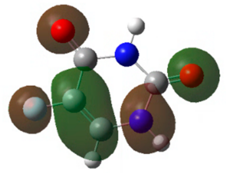	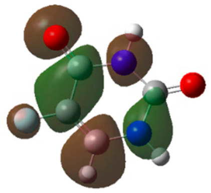	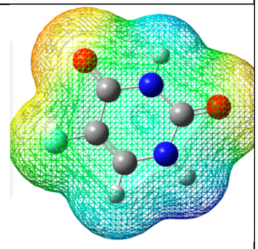
Se_6 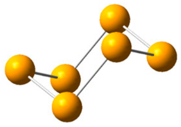	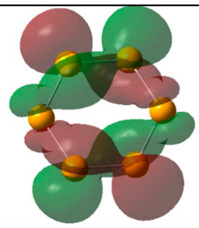	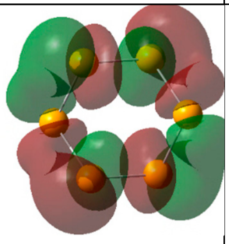	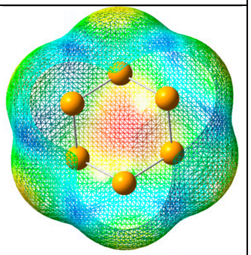
$(Se_6)_2$ 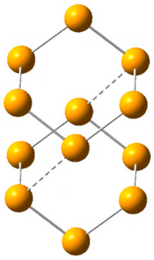	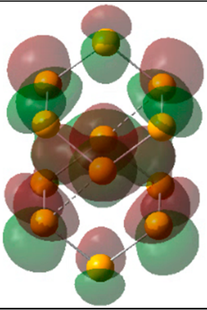	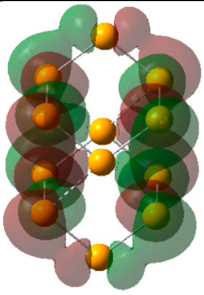	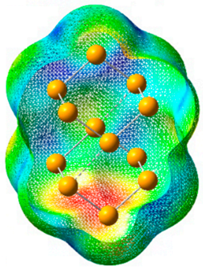

Figure S2. Optimized geometries of $Se_6/5\text{-}FU^{Ox}$, $Se_6/(5\text{-}FU)_2^{Ox}$ and $(Se_6)_2/5\text{-}FU^{Ox}$ complexes, HOMO and LUMO orbitals, and molecular electrostatic potential, obtained with B3LYP/6-31G(d,p) in a vacuum or in a gaseous medium.

Molecule	HOMO	LUMO	MEP
$Se_6/5\text{-}FU^{Ox}$ 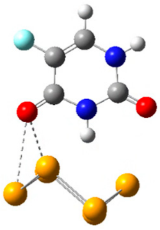	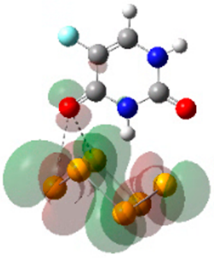	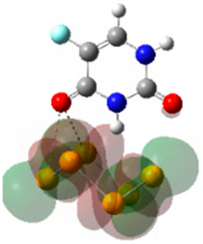	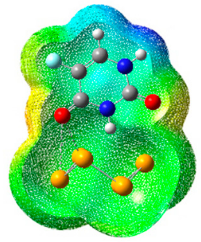
$Se_6/(5\text{-}FU)_2^{Ox}$ 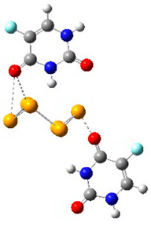	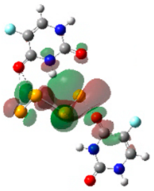	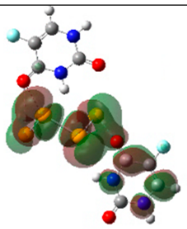	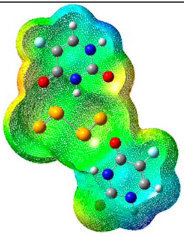

$(Se_6)_2/5\text{-}FU^{Ox}$ 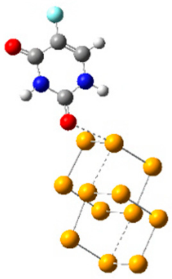	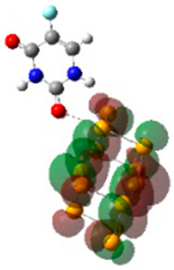	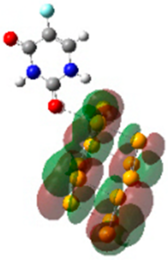	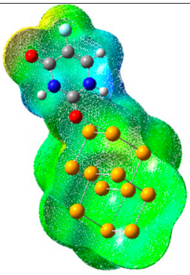

**Figure S3**. Optimized geometries of $Se_6/5\text{-}FU^{NH}$, $Se_6/(5\text{-}FU)_2^{NH}$ and $(Se_6)_2/5\text{-}FU^{NH}$ complexes, HOMO and LUMO orbitals, and molecular electrostatic potential, obtained with B3LYP/6-31G(d,p) in a vacuum or in a gaseous medium.			
Molecule	HOMO	LUMO	MEP
$Se_6/5\text{-}FU^{NH}$ 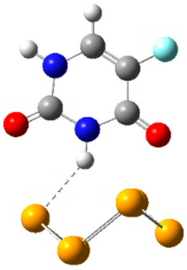	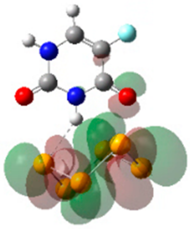	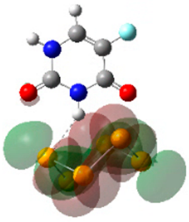	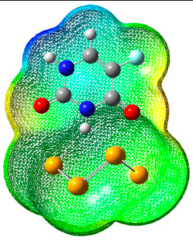
$Se_6/(5\text{-}FU)_2^{NH}$ 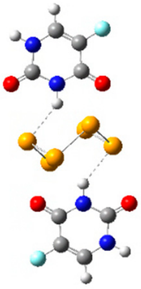	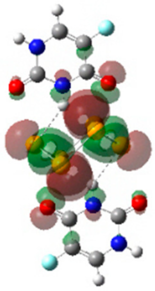	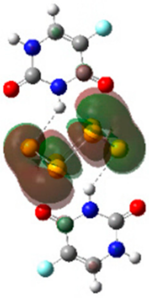	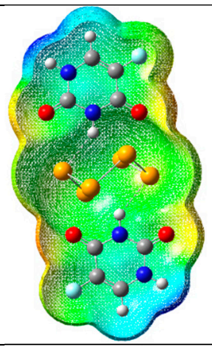
$(Se_6)_2/5\text{-}FU^{NH}$ 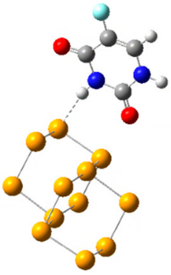	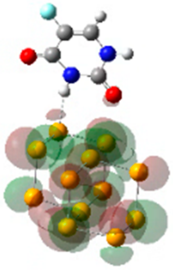	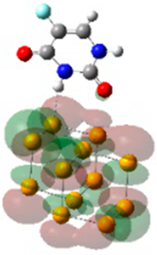	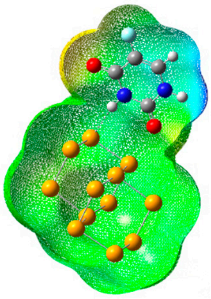

Figure S4. Optimized geometries of Se₆/5-FU^{NH}, Se₆/(5-FU)^{Ox} complexes, HOMO and LUMO orbitals, and molecular electrostatic potential, obtained with CAM-B3LYP-D3/CC-PVTZ using CPCM solvation model (water).

Molecule	HOMO	LUMO	MEP
Se_6/5-FU^{NH} 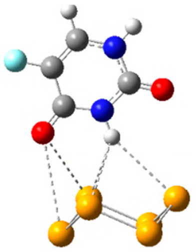	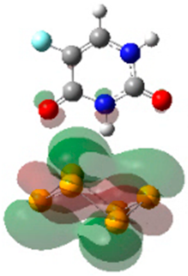	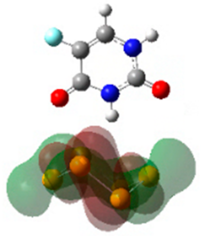	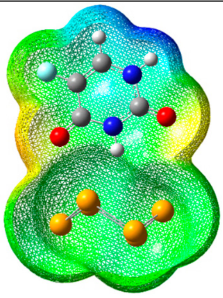
Se_6/5-FU^{Ox} 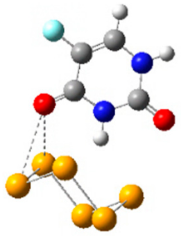	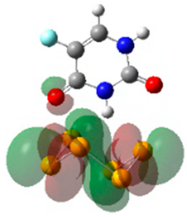	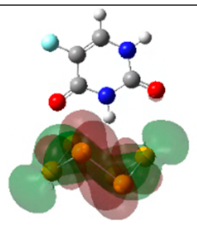	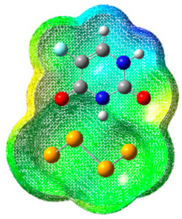

Table S1. Optimized Total Energy (E_T), Energy of the Frontier Molecular Orbitals (E_{HOMO} and E_{LUMO}), Molecular Gap Energy (Eg), and Global Molecular Descriptors (η, μ, ω) of all molecules and complexes (in aqueous media). All values in eV.

MOLECULE B3LYP/6-31G(d,p)									
Descriptor	E_T	E_{HOMO}	E_{LUMO}	E_{gap}	I	A	μ	η	ω
Se_6/5-FU^{Ox}	-406116.04	-7.9220	-1.6291	6.2929	7.9221	1.6291	-4.7756	3.1465	3.6241
Se_6/5-FU^{NH}	-406117	-7.9040	-1.4160	6.4880	7.9041	1.4161	-4.6601	3.2440	3.3472
MOLECULE CAM-B3LYP-D3/CC-PVTZ									
Se_6/5-FU^{Ox}	-405735.79	-6.3865	-2.4027	3.9837	6.3865	2.4027	-4.3946	1.9919	4.8479
% Error	0.09%	19.38%	47.49%	36.69%	19.38%	47.49%	7.98%	36.69%	33.77%
Se_6/5-FU^{NH}	-405735.74	-6.4452	-2.3595	4.0858	6.4452	2.3595	-4.4024	2.0429	4.7435
% Error	0.09%	18.46%	66.62%	37.03%	18.46%	66.62%	5.53%	37.03%	41.72%

Table S2. BSSE correction adsorption energy (E_{ads}) and cohesion energy (E_{coh}) B3LYP/6-31G(d,p). All values in eV in a vacuum or in a gaseous medium.

Complex	E_{ads}	E_{coh}
Se6/5-FUOx	1.41295191	-5.67218004
Se6/5-FUNH	1.39216240	-5.67333501
%Error Prom.	**1.785%**	**1.859%**

We have performed additional single-point calculations applying the Boys–Bernardi correction in the presence of implicit solvent by explicitly constructing fragment calculations with ghost atoms.

The corrected (BSSE) adsorption energy value is -0.4709 eV, while the uncorrected value (as shown in the original manuscript) is -0.4643 eV. This corresponds to a difference of only **0.0066 eV (~0.15 kcal/mol)**.

Such a small deviation is well below the typical accuracy of DFT methods and indicates that **BSSE has a negligible effect on the adsorption energy in this system when solvent effects are included**.

Table S3. The boys and Bernardi Correction (BSSE) whit implicit solvent water

	**uncorrected**		**BSSE corrected**	
**Molecule**	E_T **(hartree)**	E_T **(eV)**	E_T **(hartree)**	E_T **(eV)**
**Se6**	**-14396.4298**	**-391746.9951**	**-14396.4296**	**-391746.9903**
**5FU**	**-514.0597**	**-13988.2826**	**-514.0596**	**-13988.2807**
**Se6/5-FUNH**	**-14910.5065**	**-405735.7419**	**-14910.5065**	**-405735.7419**
E_{ads} **(eV)**	**-0.4643**		**-0.4709**	

Figure S5 shows the molecular models followed to perform the calculations in Table S3.

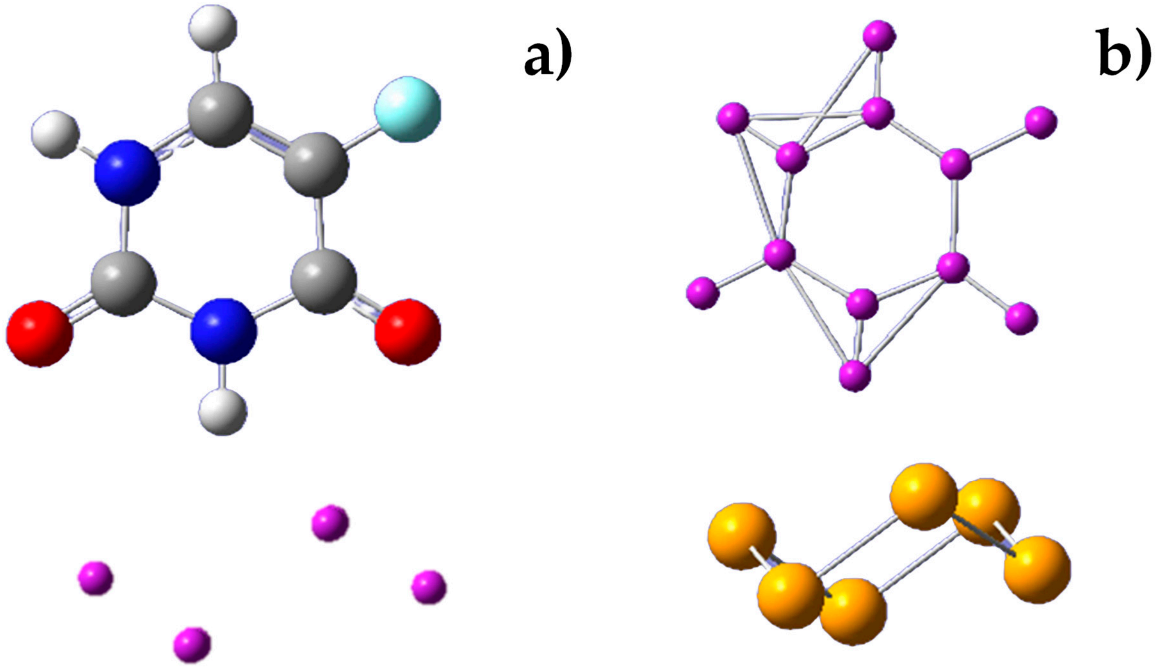

Figure S5. File construction for the correction the boys-Bernardi a) 5-FU molecule in ground state and Se_6 in ghost state, b) Se6 in ground state and 5-FU in ghost state.

Table S4. Enthalpy, Gibbs free energy and entropy (kcal/mol) and dipole moment (Debyes) of the molecular complexes in a vacuum or in a gaseous medium.

Reaction	ΔH	ΔG	TΔS	$\vec{\mu}(D)$
$(Se_6 + 5\text{-FU} \rightarrow Se_6/5\text{-FU})^{Ox}$	-12.5853	-1.0919	-11.4935	3.9038
$(Se_6 + 2\text{-}5\text{-FU} \rightarrow Se_6/(5\text{-FU})_2)^{Ox}$	-22.1655	1.6497	9.4804	3.3880
$(2Se_6 + 5\text{-FU} \rightarrow (Se_6)_2/5\text{-FU})^{Ox}$	-5.0169	9.4804	-14.4974	4.2104
$(Se_6 + 5\text{-FU} \rightarrow Se_6/5\text{-FU})^{NH}$	1.2374	13.7155	-12.4780	4.4549
$(Se_6 + 2\text{-}5\text{-FU} \rightarrow Se_6/(5\text{-FU})_2)^{NH}$	-26.8273	-0.4938	-26.3334	6.8615
$(2Se_6 + 5\text{-FU} \rightarrow (Se_6)_2/5\text{-FU})^{NH}$	-5.0752	7.4804	-12.5556	3.9876

To low-frequency vibrational modes (<100 cm^{-1}) may lead to an overestimation of entropy and, consequently, affect the computed Gibbs free energies within the standard rigid-rotor harmonic oscillator (RRHO) approximation. To evaluate this effect, we performed a quasi-harmonic correction for a representative system, Se_6/5-FU^{NH}, using a frequency cutoff of 100 cm^{-1}. This approach replaces low-frequency modes with a minimum threshold, thereby reducing the artificial entropic contribution associated with very soft vibrations. The correction was carried out using the GoodVibes program [74], which implements the quasi-harmonic approximation following the Grimme entropy treatment and Head-Gordon enthalpy correction [75].

The uncorrected Gibbs free energy change (ΔG) was found to be 15.9657 kcal/mol (as shown in manuscript, Table S5, entry 4), while the quasi-harmonic corrected value is 15.8653 kcal/mol, corresponding to a difference of **only 0.10 kcal/mol**. This very small deviation indicates that low-frequency vibrational modes have a negligible impact on the computed thermodynamic quantities in this system. Therefore, the use of the standard RRHO approximation does not affect the reliability of the reported energetic trends or the conclusions of this work.

Table S5. Corecction of freee energy Gibss >-100 frequency

Molecule	**G (RRHO) / hartree**	**G (qh) / hartree**
5-FU	-514.012364	-514.012545
Se_6	-14396.46463	-14396.46521
Se_6/5-FU^{NH}	-14910.45155	-14910.45247
$\Delta G\ (hartree)$	0.025283	hartree
$\Delta G\ (kcal \cdot mol^{-1})$	**15.8653254**	**kcal/mol**
